# The serial blocking effect: a testbed for the neural mechanisms of temporal-difference learning

**DOI:** 10.1038/s41598-019-42244-4

**Published:** 2019-04-12

**Authors:** Ashraf Mahmud, Petio Petrov, Guillem R. Esber, Mihaela D. Iordanova

**Affiliations:** 1 Department of Psychology, Center for Studies in Behavioral Neurobiology/Groupe de recherche en neurobiologie comportementale, Concordia University, Montreal, Quebec, Canada; 2 Department of Psychology, Brooklyn College of the City University of New York, Brooklyn, NY USA

## Abstract

Temporal-difference (TD) learning models afford the neuroscientist a theory-driven roadmap in the quest for the neural mechanisms of reinforcement learning. The application of these models to understanding the role of phasic midbrain dopaminergic responses in reward prediction learning constitutes one of the greatest success stories in behavioural and cognitive neuroscience. Critically, the classic learning paradigms associated with TD are poorly suited to cast light on its neural implementation, thus hampering progress. Here, we present a serial blocking paradigm in rodents that overcomes these limitations and allows for the simultaneous investigation of two cardinal TD tenets; namely, that learning depends on the computation of a prediction error, and that reinforcing value, whether intrinsic or acquired, propagates back to the onset of the earliest reliable predictor. The implications of this paradigm for the neural exploration of TD mechanisms are highlighted.

## Introduction

Error-correcting algorithms as specified by associative (e.g.)^1^ and temporal-difference reinforcement learning (TDRL; e.g.)^2^ models have provided a particularly useful theory-driven approach to examining how learning is implemented in the brain. Indeed, uncovering the neural signature of TDRL has been at the forefront of brain science for decades. However, the extent to which TDRL’s two fundamental assumptions can be dissected at the neural level has been limited by the traditional learning paradigms available.

The first assumption is that reinforcement learning is driven by a prediction error, or difference between the expected and experienced outcome. This is assessed using the blocking paradigm, in which a cue fails to become a predictor of an outcome if it is trained in the presence of a good predictor for the same outcome (thus making the prediction error equal to zero)^3,4^. Blocking has been a cornerstone paradigm in neuroscience, delivering a series of key findings on the neurobiological mechanisms of learning in fear and reward^5–11^. But despite its importance, the classic design has shortcomings that have limited its application to neuroscience, particularly in the context of temporally precise neuronal recording (e.g., behavioural electrophysiology) or manipulation techniques (e.g., optogenetics). This is because in the standard design the blocking and blocked cues are presented simultaneously in compound, thus making it difficult to individually track neural responses to each cue as well as to dissociate the effects of neural manipulations on them. For instance, neural dynamics linked to the setting of reinforcement expectancies by the blocking cue may be confounded with those underpinning attentional changes based on novelty to learning to either cue during the compound phase.

The second cardinal assumption of TDRL is that during training the prediction error gradually propagates back to the onset of the earliest reliable predictor of reinforcement, imbuing every instant in between with varying degrees of reinforcing properties. A behavioural paradigm that is apt for examining this tenet at the neural level is second-order conditioning, but it also carries drawbacks from the behavioural and neuroscience standpoints. Second-order conditioning, where a target cue is never directly paired with reinforcement but with another cue that was previously reinforced, only yields transient conditioning as the target cue soon becomes a signal for reinforcement omission (i.e., a conditioned inhibitor)^12^.

To provide a more suitable testbed for examining TDRL’s tenets and their neural underpinnings, we designed a serial blocking paradigm in which the blocking and blocked cues are serially presented during the blocking phase. In this design, the blocking cue is initially trained in a trace conditioning procedure in which cue offset and reinforcer onset are separated by a trace interval (blocking cue → trace → reinforcer). In the blocking phase, the target, to-be-blocked cue is introduced during the trace interval (blocking cue → blocked cue → reinforcer). We found that serially presenting the cues yielded an equivalent amount of blocking to that observed in the standard blocking design. Moreover, in the serial control condition we observe an additive effect of second-order conditioning of the earliest cue in the sequence, superimposed on first-order conditioning. As will be seen, these findings have important implications for the neural exploration of reinforcement learning mechanisms.

## Serial cue presentation provides an effective blocking examination

**Figure 1:**
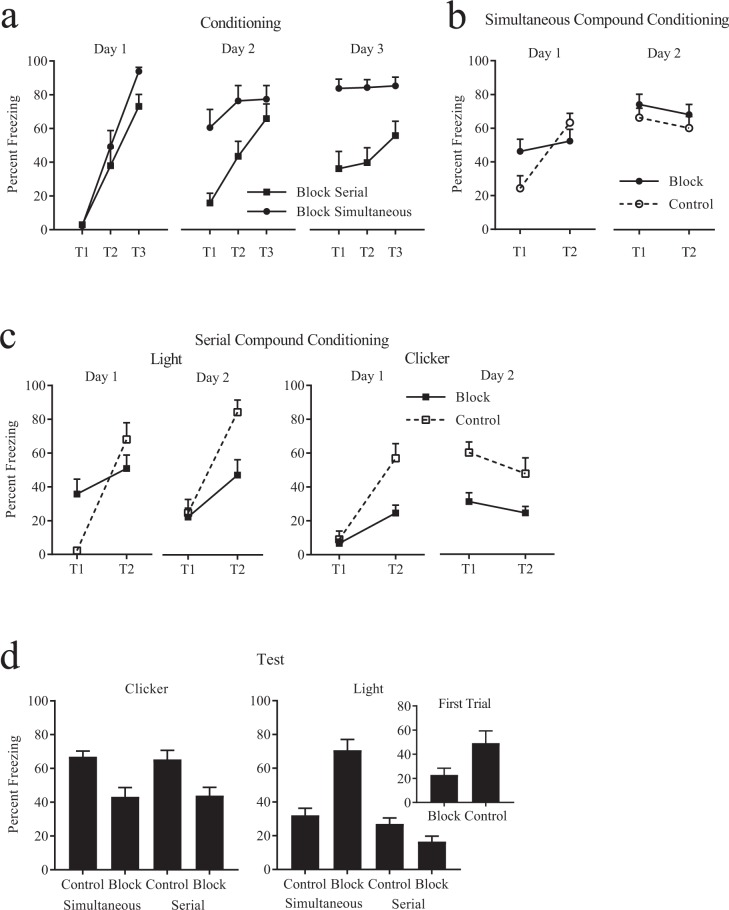
Serial cue presentation provides an effective blocking examination. (**A**) Increase in freezing levels in Groups Block Serial and Block Simultaneous during Conditioning. Fear to the pre-trained cue in Group Simultaneous was greater than that of Group Serial. (**B**) Simultaneous compound conditioning in Groups Control Simultaneous and Block Simultaneous revealed an increase in freezing on Day 1 and a maintenance of this high level of fear on Day 2. (**C**) Serial compound conditioning in Groups Control Serial and Block Serial revealed a blocking effect from the second trial of Day 1, which was then maintained on Day 2 with Group Control showing consistently higher levels of freezing to the clicker compared to Group Block (F_1,22_ = 11.8, CI [0.46, 1.85]). (**D**) Freezing to the clicker was lower in the blocking groups compared to the control groups and the effect of blocking was similar between the compound and serial groups. Freezing to the pre-trained light was greater in Group Block Simultaneous compared to Control Simultaneous, but the direction of this difference was reversed for the serial groups at least on the first trial (inset).

During *Conditioning* rats in Group Block Simultaneous received delay pairings between a light and a shock whereby cue offset coincided with shock onset, whereas rats in Group Block Serial received trace conditioning whereby a 30 s interval was interpolated between cue offset and shock onset. As expected (see Fig. 1a), fear indexed by freezing increased across days in both groups (F_1,22_ = 27.5, CI [0.68, 15.6]), with greater levels of freezing reported in the delay compared to the trace procedure (F_1,22_ = 19.3, CI [0.76, 2.12]), with the former acquiring this fear at a faster rate (F_1,22_ = 14.2, CI [0.72, 2.49]). Subsequently, during *Blocking*, all rats received two presentations of the pre-trained light and a novel clicker either in compound (i.e., concurrent light and clicker, Groups Block Simultaneous and Control Simultaneous) or serially (light followed by the clicker, Groups Block Serial and Control Serial) with each presentation terminating in shock. Freezing to the compound in Groups Block Simultaneous and Control Simultaneous (see Fig. 1b) differed on the first trial of Phase 2 training, but disappeared by the second trial, revealed by no effect of group (F_1,22_ < 1, CI [−0.49, 0.95]), a linear trend across trials (F_1,22_ = 19.1, CI [0.50, 1.41]) and an interaction (F_1,22_ = 10.1, CI [−2.29, −0.48]). An identical analysis revealed no effects on the second day of Phase 2 (maxF_1,22_ = 3.7, CI [−0.53, 0.02]). Fear to the light (Fig. 1c: Light) and clicker (Fig. 1c: Clicker) were examined separately in the serial groups. Freezing to the light in Groups Block Serial and Control Serial differed on the first trial of Day 1 in Phase 2 training but this difference disappeared by the second trial, revealed by no effect of group (F_1,22_ < 1, CI [−0.36, 0.96]), a linear trend across trials (F_1,22_ = 34.1, CI [0.97, 2.04]) and an interaction (F_1,22_ = 13.3, CI [−2.95, −0.81]). Interestingly, by the end of the second day of Phase 2, Group Control Serial showed a higher level of fear to the light compared to Group Block (F_1,22_ = 6.2, CI [0.13, 1.39]) despite the latter having received a greater number of light-shock pairings overall (i.e., Phase 1 and Phase 2). Freezing to the clicker proceeded as expected for Groups Block Serial and Control Serial. There was an increase in fear across trials (F_1,22_ = 29.2, CI [1.04, 2.33]), with a greater increase seen in Group Control Serial compared to Block Serial (an effect of group: F_1,22_ = 11.3, CI [0.34, 1.45], and an interaction: F_1,22_ = 6.0, CI [0.24, 2.82]). Thus blocking could be observed immediately following learning on the first trial of serial compound conditioning. This affords a trial-based theoretical examination of the mechanisms that drive blocking^1,13,14^ and shows that blocking results from a downregulation of outcome processing^1^ as opposed to cue/attentional processing^13,14^ in the present case. This blocking effect was maintained on the following day (see Fig. 1 legend for statistics). The serial blocking design offers an online confirmation of the effectiveness of blocking, which eliminates the disruptive effects of testing under conditions different to those of acquisition, including but not limited to any perceptual or behavioural masking of the novel cue by the pre-trained cue illustrated in the simultaneous compound. Further, this effect provides evidence against any role for local contextual cues on blocking^15^ or we would see high levels of fear to the novel cue in Phase 2.

The groups received non-reinforced *Tests* to the individual stimuli (Fig. 1d). Freezing to the clicker in the Control groups was higher compared to the Block groups (see Fig. 1d; F_1,44_ = 22.2, CI [0.78, 1.94]) and there was no effect of compound presentation (F_1,44_ < 1, CI [−0.56, 0.61]) nor was there an interaction (F_1,44_ < 1, CI [−0.65, 0.51]), thus providing evidence that blocking can be obtained with a serial and a simultaneous procedure. Freezing to the light was higher in Group Block Simultaneous compared to Group Control Simultaneous (F_1,44_ = 37.1, CI [1.67, 3.31]). This was expected, as the light was pre-trained in the case of Group Block but not in the case of Group Control. This difference in freezing to the light between the Block and Control groups was not seen in the Serial training conditions (Block Serial vs. Control Serial : F_1,44_ = 2.8, CI [−1.50, 0.15]). Therefore, we examined the level of freezing to the light on the *first trial* of Test before non-reinforcement could mask differences between the groups. Freezing to the light on the *first trial* of Test (Fig. 1d inset) in Group Control Serial was higher than Group Block Serial (F_1,44_ = 6.3, CI [1.84, 0.20], but as expected, the direction of this difference was reversed for Groups Control Simultaneous and Block Simultaneous, F_1,44_ = 17.0, CI [−0.86, −2.51], data not shown). The higher level of freezing to the light in Group Control Serial compared to Group Block Serial seems counterintuitive given the extra conditioning trials the latter group received during Phase 1. However, conditioning to the pretrained cue is relatively weak due to the trace period between cue offset and shock onset. Furthermore, fear to the first cue (light) in the serial compound is determined not only by the direct association of that cue with the shock, but also by the backpropagation of the association of the second cue (clicker) in the serial compound with the shock, i.e., second-order conditioning. As noted earlier, conditioning to the second cue (clicker) is greater in Group Control Serial compared to Group Block Serial (i.e., the blocking effect), which results in higher levels of second-order conditioning in the former compared to the latter group (see also)^2,16^.

## Second-order conditioning in a serial compound procedure

**Figure 2:**
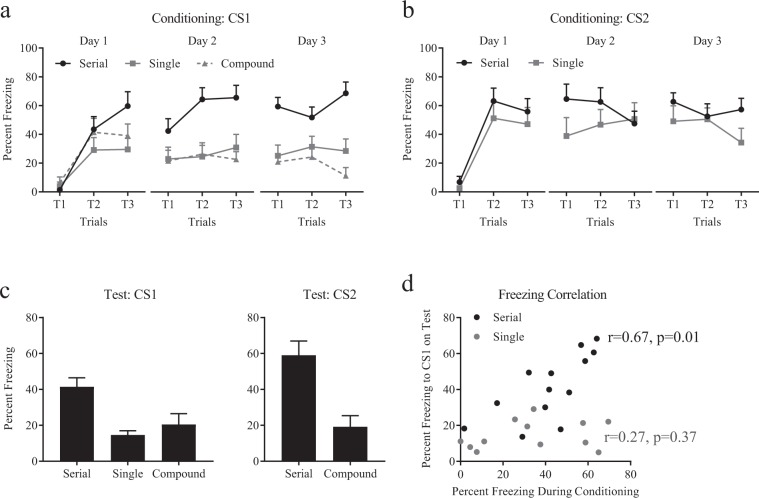
Second-order conditioning in a serial compound procedure. (**A**) Fear to CS1 increased faster in Group Serial compared to Groups Single and Compound on Day 1. On subsequent days of conditioning, fear to Group Serial remained higher compared to the other two groups individually (Day 2: Single F_1,26_ = 14.14, CI [0.42, 1.82]; Compound F_1,26_ = 16.62, CI [0.52, 1.91]; Day 3: Single F_1,26_ = 16.28, CI [0.53, 1.99]; Compound F_1,26_ = 29.98, CI [0.96, 2.39]), steady (Day 2: F_1,26_ = 3.43, CI [−0.04, 0.80]; Day 3: F < 1, [−0.37, 0.44]) with no interactions (Day 2: Serial vs. Single F_1,26_ = 1.16, CI [−0.64, 1.72]; Serial vs. Compound F_1,26_ = 2.47, CI [−0.39, 1.97]; Day 3: Serial vs. Single F < 1, [−0.92, 1.40]; Serial vs. Compound F_1,26_ = 2.50, CI [−0.37, 1.90]). (**B**) Fear to CS2 in Group Serial and the equivalent temporal interval post CS1 in Group Single increased on the first day but did not differ between the groups on any of the days (see main text for Day1). There were no differences between the groups (Day 2: F_1,18_ = 1.04, CI [−0.35, 1.04]; Day 3: F_1,18_ = 1.67, CI [−0.25, 1.09]), no linear trend across trials (Day 2: F < 1, [−0.54, 0.40]; Day 3: F_1,18_ = 2.50, CI [−0.77, 0.10]) and no interaction (Day 2: F_1,18_ = 2.94, CI [−1.71, 0.16]; Day 3: F < 1, [−0.56, 1.18]). (**C**) Freezing to the cue during non-reinforced tests show that fear to CS1 was greater in Group Serial compared to Groups Single and Compound, and freezing to CS2 was greater in Group Serial compared to Group Compound. (**D**) The overall levels of freezing to CS2 during conditioning were correlated with freezing to CS1 during test.

In this second experiment we sought to confirm the elevated conditioning seen to the first cue of a serial compound and explore the role of the second cue in this learning. Three groups of rats were conditioned such that Group Serial received two sequential cues (conditioned stimuli, CSs)  of different modalities (visual and auditory, counterbalanced) where CS1 offset coincided with CS2 onset, and CS2 offset coincided with shock onset (CS1 → CS2 → shock); Group Single received trace conditioning with CS1 only (CS1 → trace → shock); Group Compound received trace conditioning with CS1 and CS2 presented in compound (CS1&CS2 → trace → shock). Stimuli were presented and analyzed in accordance with their temporal relationship with shock. During *Conditioning* on Day 1, fear to CS1 (Groups Serial and Single) and CS1 + CS2 (Group Compound) increased across trials (Fig. 2a; F_1,26_ = 58.04, CI [1.09, 1.88]), with no group differences (Serial vs. Single: F_1,26_ = 3.35, CI [−0.15, 1.21]; Serial vs. Compound: F < 1, CI [−0.46, 0.90]), but an interaction between Groups Serial and Single (F_1,26_ = 6.94, CI [0.14, 2.36]) revealing a greater rate of increase for Group Serial compared to Group Single, but not for Groups Serial vs. Compound (F_1,26_ = 4.21, CI [−0.14, 2.09]). On the subsequent conditioning days, fear to CS1 was higher in Group Serial compared to the other groups (see Fig. 2 legend for statistics).

CS2 was presented following CS1 in group Serial, therefore the best comparison for CS2 was the equivalent temporal interval following CS1 presentation in Group Single. Fear to CS2 in Group Serial (Fig. 2b) was similar to fear during the same temporal interval following CS1 offset in Group Single (F < 1, CI [−0.32, 0.87]). This fear increased across trials (F_1,18_ = 40.89, CI [1.05, 2.06]) but did not do so differentially between the two groups (F < 1, CI [0.86, 1.16]). No statistical differences were detected between the two conditions on subsequent days (see Fig. 2 legend).

Rats were tested for fear to the CS1 and CS2 (where applicable) during non-reinforced sessions. Freezing to CS1 (i.e., Primacy cue, Fig. 2c: CS1) was greater in Group Serial compared to Groups Single and Compound (F_1,27_ = 14.42, CI [0.60, 1.99]), while the latter two groups did not differ from one another (F < 1, [−1.00, 0.63]). These data provide evidence for a primacy effect when cues are presented serially. There was no effect of training with a single or a compound CS on learning about CS1 in trace conditioning. Freezing to CS2 (Fig. 2c: CS2) was greater in Group Serial compared to Group Compound (t_19_ = 3.64, p = 0.001), due to the closer temporal position of CS2 to footshock in Group Serial compared to Group Compound. Interestingly, fear to CS2 during conditioning (Days 1–3) predicted fear to CS1 on test for Group Serial (r = 0.674, p = 0.012) but fear during the same temporal interval in Group Single did not predict fear to CS1 on test (r = 0.273, p = 0.366; Fig. 2d). These data show that training in a serial compound results in stronger conditioning to the first reliable predictor of the compound; that is, fear propagated back to CS1. Furthermore, our data show that this is dependent on the presence of a ‘bridging’ (CS2) stimulus between CS1 offset and US onset, and that the amount of conditioning acquired by this bridging stimulus across training predicts the amount of fear that is expressed by the temporal primacy stimulus (CS1) on test. In other words, the associative strength acquired by CS2 transferred to CS1 akin to second-order conditioning.

## Discussion

In this article, we presented a serial blocking paradigm that is specifically designed to explore the neural circuits underpinning TDRL. This paradigm is ideally suited to investigating the neural bases of TDRL’s fundamental assumptions that (1) learning will not occur in the absence of a prediction error and that (2) the value of the reinforcer propagates back to the onset of the earliest reliable predictor via the second-order conditioning effect observed in Group Control Serial. Particularly noteworthy is the fact that, unlike in second-order conditioning, the effect observed in the serial control group does not compete with the development of conditioned inhibition^12^. This is a critical advantage in single-unit recording studies where a high number of training trials is desirable.

In addition to being able to test both assumptions at once, the current paradigm offers the neuroscientist the advantage of temporally uncoupling the presentation of the blocking and blocked stimuli. This allows for a dissociable examination of the contribution of specific neural circuits to cognitive processes related to each of these cues. For instance, one could optogenetically target neural structures implicated in the generation of reinforcement expectancies by the blocking cue (e.g., basolateral amygdala or prelimbic cortex in fear^17–19^, orbitofrontal cortex in reward)^20,21^ without affecting redundancy-driven decrements in the salience of the blocked cue^9^. Similarly, one could separately examine the contribution of mesolimbic dopamine to (1) temporally specific predictions set up by the blocking cue, (2) prediction error at the time of reinforcement^22^, and (3) novelty-related salience when the blocked cue is first introduced (e.g.)^23–26^. While the present design focuses on fear, staggered but still overlapping presentations of the pre-trained cue and the blocked cue have been effective in producing blocking with a rewarding outcome^27,28^, thus leaving no reason to suppose that the current serial design would be ineffective in the appetitive setting. Thus, in combination with techniques with high temporal resolution such as single-unit recording and optogenetics, the serial blocking paradigm offers an unprecedented opportunity to dissect the reinforcement learning circuit.

Notably, the above advantages over the simultaneous paradigm come at no cost in terms of the strength of the blocking effect. The equivalent size of the blocking effect in the serial and simultaneous blocking groups presumably reflects a comparable expectation of reinforcement at the time of its delivery despite the lower level of responding to the blocking cue observed in the serial group. Thus, the serial blocking paradigm allows the neuroscientist to dissociate a predictor’s ability to evoke conditioned behavior (e.g., freezing) from its ability to generate temporally-precise reinforcement expectancies and produce blocking. This is consistent with a dissociation between the acquisition of value and that of temporally-precise reinforcement expectancies, as specified by^29^ as well as the predicted-time-of-arrival hypothesis^30^. Finally, our paper together with the existing body of literature provide procedural guidance in obtaining blocking. Specifically, blocking will be observed when cue arrangements maintain a consistent temporal relationship between the pre-trained cue and the outcome across phases^31–33^ irrespective of cue length^27^, when the *blocking cue* precedes the outcome^32^, and the delivery of the novel cue does *not* precede the pre-trained cue^16,27,31,32^.

## Materials and Methods

### Subjects

Forty-eight Long-Evans rats (Charles River; St. Constant, Quebec, Canada) were used (12 rats per group, equal number of males and females) in Experiment 1. Thirty seven rats (21 males and 16 females) of Long-Evans background (*M* = 347.5 ± 10.16 g) were used in Experiment 2. One rat (Group Single) was excluded from the analyses of Experiment 2, because it was deemed to be an outlier according to the Grubb’s outlier test (Zc = 2.46 Z = 2.57 https://www.graphpad.com/quickcalcs/Grubbs1.cfm). The weights of the rats ranged between 275 and 325 g at the beginning of the experiments. All rats had *ad libitum* access to food and water and were housed in pairs in standard clear shoebox cages in a humidity and temperature-controlled environment under reverse light-dark conditions (12:12 h light-dark cycle; lights off at 8:00 a.m.), and with experimental sessions occurring about 3–4 hours after the onset of the dark cycle. Rats were handled once a week during the acclimation period in the colony and then daily for 3 days prior to the experiments. All rats were treated in accordance with the approval granted by the Canadian Council on Animal Care and the Concordia University Animal Care Committee.

### Apparatus

Behavioral sessions were conducted in standard operant-training chambers (Med Associates, St. Albans, VT, USA). The chambers measured 31.8 cm in length, 25.4 cm in width and 26.7 cm in height and were enclosed in ventilated wooden compartments, which provided approximately 50 dB background noise in the chambers. Each chamber was comprised of a stainless-steel grid floor, modular left and right walls, and Perspex back wall, front door and ceiling. The grid floor was connected to a shock generator and delivered continuous scrambled footshock. The left wall housed two white cue lights (28 V DC, 100 mA stimulus light) located 15 cm below the ceiling on the left and right panels; a red house light (28 V DC, 100 mA stimulus light with red replacement lens cover) located 5 cm below the ceiling on the centre panel; and mechanical clicker located below the red house light. A computer running Med PC IV (Med Associates) software on Windows OS controlled the experimental protocols. All sessions were videotaped.

### Stimuli

The auditory stimulus used in all three experiments was a 30 s 10 Hz 75 dB mechanical clicker and the visual stimulus was a 30 s 20 Hz light located on the left-hand side of the right panel. The somatosensory unconditioned stimulus was a 0.5 mA 1 s footshock.

### Behavioral Procedures

Experiment 1 consisted of 4 phases: habituation, conditioning during Phase 1 and Phase 2, and non-reinforced Tests. Experiment 2 consisted of 3 phases: habituation, conditioning and non-reinforced Tests.

#### Habituation

On Day 0 rats were habituated to the auditory and visual stimuli. The habituation session lasted one day and consisted of two presentations of each cue (clicker or flashing light) 5 min upon placement in the experimental chambers. The cues were presented two times each for 30 s with an intertrial interval (ITI) of 2 min and the session lasted for a total of 16 min.

#### Experiment 1: Phase 1 Conditioning

On each of Days 1–3, rats in the Blocking groups received three pairings between the flashing light and shock for a total of nine such pairings across Phase 1. The first light-shock pairing took place 5 min upon placement in the conditioning chamber, and successive pairings were separated by an average of 5 min ITI (range: 240–360 s). The last light-shock pairing occurred 4.5 min prior to the end of the training session. Rats were brought and placed in the operant chambers again 3.5 hours after the training sessions in order to receive exposure to the context (no cues or other stimuli presented) to reduce freezing to the background cues. Rats in the Control groups did not receive Phase 1 conditioning and were merely handled outside the laboratory.

#### Experiment 1: Phase 2 Blocking

On Days 4 and 5, all rats received compound conditioning. Compound conditioning consisted of two presentations of the flashing light and clicker in compound paired with shock, for a total of four such pairings across Phase 2. For a description of the relationship between the cues (CSs) and the shock (unconditioned stimulus, US) see section ‘Experiment1: CS-US relation’ below.

#### Experiment 1: CS-US relation

For rats in the Simultaneous groups (Block Simultaneous and Control Simultaneous) the CS (or CSs) were trained in a delay procedure such that shock (unconditioned stimulus, US) onset coincided with CS offset, i.e., the cues were presented for 30 s at the end of which a shock was delivered. For rats in the Serial groups (Block Serial and Control Serial) the light CS was trained in a serial fashion such that light offset was followed by a 30 s trace period at the end of which the shock was delivered (Phase 1) or light offset coincided with clicker onset, and clicker offset coincided with shock onset (Phase 2).

#### Experiment 2: Conditioning

Phase 1 lasted 3 days. Rats in Group Serial received conditioning trials in a serial delay procedure such that CS1 offset coincided with CS2 onset and CS offset coincided with shock onset. For rats in Group Single, a single CS, i.e., CS1, was paired with the shock with an interval between CS offset and shock onset of 30 s. For rats in Group Compound, a simultaneous compound presentation of CS1 and CS2 was paired with the shock with an interval between compound offset and footshock onset of 30 s. All groups received three pairings per day, for a total of nine pairings across Phase 1. The first pairing took place five minutes upon placement in the conditioning chamber, and successive pairings were separated by an average ITI of 5 min (range: 240–360 s). The last CS-shock pairing occurred four minutes prior to the end of the training session. Rats were brought and placed in the operant chambers again 3.5 hours after the training sessions in order to receive exposure to the context (no cues or other stimuli presented) to reduce freezing to the background cues.

#### Experiment 1: Non-reinforced Tests

Rats were tested for fear to the clicker on Days 6 and 7 and to the flashing light on Day 8. Data for fear to the clicker were pooled between the two tests. Test sessions consisted of eight 30 s nonreinforced presentations of the conditioned cues (light or clicker) 1 min apart. Each test session consisted of a 5 min acclimation period prior to the first presentation of a cue. Rats were removed from the conditioning chambers 1 min following the last (eight) presentation of the cue.

#### Experiment 2: Non reinforced Tests

Rats were tested for fear to CS1 and CS2 on Days 6 and 7 respectively (i.e., rats in Group Single were only tested to the conditioned cue on Day 6). Procedurally, the Test sessions were identical to those described in Experiment 1.

### Scoring and Statistics

All sessions were videotaped and scored offline. Freezing behavior was scored on a second-by-second basis with a timestamp procedure in which each rat was observed for the entire session and scored as either freezing or moving. Freezing was defined as the absence of all movement, except for those related to breathing (R. J. Blanchard & Blanchard, 1969)^34^. A percentage of the time spent over the total observation time was calculated for each rat. A second scorer blind to the subjects’ group assignment scored a random subset of the data. The correlation between the scorers (AM and PP) was 0.99. Experiment 1 was based on a classic blocking design and Experiment 2 was based on data obtained in Experiment 1, and therefore the hypotheses with regard to the directionality of the differences were pre-determined. Therefore, our data were analyzed using planned orthogonal contrasts (version 21, PSY2000). Significance was set at the 0.05, and confidence intervals were standardized and presented in standard deviation units.

## Data Availability

All data will be made available upon request.
